# GC-MS analyses and bioactivities of essential oil obtained from the roots of *Chrysopogon zizanioides* (L.) Roberty cultivated in Giresun, Turkey

**DOI:** 10.3906/kim-2009-64

**Published:** 2021-07-09

**Authors:** EFE Derya, Mustafa KARAKÖSE, Ayça AKTAŞ KARAÇELİK, Bengü ERTAN, Mehmet Emin ŞEKER

**Affiliations:** 1 Department of Medicinal and Aromatic Plants, Giresun University, Giresun, Turkey; 2 Department of Food Processing, Giresun University, Giresun, Turkey; 3 Department of Occupational Health and Safety, Giresun University, Giresun, Turkey

**Keywords:** Vetiver, essential oil, GC-MS, antimicrobial, antioxidant

## Abstract

The essential oils (EOs) constituents have trade importance due to their bioactivities. In this research, the essential oil of the roots of *Chrysopogon zizanioides* (L.) Roberty cultivated in Giresun was obtained by hydrodistillation. Then, the chemical composition and biological activities of *Chrysopogon zizanioides* root essential oil (CEO) were studied. Thirty compounds were identified by gas chromatography-mass spectrometry (GC-MS) and the major compounds were determined as isobutyl-angelate (11.15%), **α-**muurolene (10.56%), α-cedrene (8.42%), α-patchoulene (8.10%), and ethylene brassylate (7.49%). The antimicrobial effect of CEO was investigated against pathogenic bacteria and fungus. Minimal inhibition concentration (MIC) was determined 31.25 μg/mL against *C. albicans* and between 62.5 and 125 μg/mL against the rest of the microorganisms. According to the antioxidant assays, the total phenolic content of CEO was found to be 13.70 ± 2.10 μg/mL gallic acid equivalent (GAE) and 24.67 ± 3.79 μg/mL catechin equivalent (CE). Ferric reducing/antioxidant power (FRAP) of CEO was found to be 356.44 ± 2.34 μM Trolox equivalent antioxidant capacity (TEAC). 2,2-diphenyl-1-picrylhydrazyl (DPPH•) was determined as IC_50_ to be 7.124 ± 0.076 μg/mL. According to the obtained results, CEO has significant antimicrobial and antioxidant activities. This study is the first report determining the chemical composition, antimicrobial and antioxidant activities of essential oil obtained from vetiver grown in the Black Sea Region of Turkey.

## 1. Introduction

Essential oils, secondary metabolites, are produced by plants during stress periods caused by different external agents [1]. The plants synthesize aromatic and complex compounds including hydrocarbons, alcohol, aldehydes, esters, ethers, ketones, oxides, phenols, and terpenes. The essential oils (EOs) have trade importance due to their bioactivities caused by their constituents. According to the literature, EOs can be obtained from about 3000 plant species and 300 of the EOs have commercial sense [2].

The essential oil of Chrysopogon zizanioides (L.) Roberty (CEO) is commonly defined as tranquility oil and has significant commercial importance because of its unique fragrance not provided by any other synthetic compounds [3]. The yearly World trade of CEO is presumed to be about 250 tons (approximately $ 20–200 million per annum). As the main producers of CEO are Haiti, China, Japan, etc. and, the main consumers are the USA, Europe, India, and Japan [3]. CEO is extracted from Chrysopogon zizanioides (L.) Roberty belonging to the family of Poaceae. Chrysopogon zizanioides (L.) Roberty, has been used to prevent soil erosion and rehabilitate lands contaminated with heavy metals because of its extremely ever-lengthening roots and its tolerance to elevated metal levels [4]. In addition, CEO is commonly used in foods, perfumery, and medicine [5,6] for the treatments of ulcers, fever, headache, inflammation, gastritis due to functional ingredients and fragrance[5–7]. This plant taxon has been naturally grown in India and extensively cultivated in the tropical regions of the World [7]. Many researchers have studied chemical compositions and bioactivities of CEO, which has already been used by local people for different purposes for hundreds of years in different regions [3,4]. Vetiver grass was brought to Turkey from Nepal in 1998 and cultivated for the first time in Çoruh Basin (Artvin province) and then in Maçka (Trabzon province) to prevent severe erosion of the region [8]. However, there has been no study about chemical compositions and bioactivities of vetiver grown in Black Sea Region of Turkey except the study reported the antibacterial effect of CEO only against four bacteria [9]. According to previous researches, the chemical compositions and bioactivities are mostly affected by many factors such as geographical, climatic, seasonal, experimental conditions, water stress, and origin, the chemo-type, chemical polymorphism, and the stage of the plants [10]. In this regard, it is of great importance to determine the chemical composition and bioactivities of the CEO obtained from the roots of vetiver cultivated in Tirebolu/Giresun located in Black Sea Region of Turkey.

In this study, the compounds of the hydrodistilled CEO were identified by GC-MS analyses. Then, the antimicrobial and antioxidant activities were identified by disc diffusion, minimal inhibition concentration assays, and 2,2-diphenyl-1-picrylhydrazyl (DPPH•) radical scavenging assay, ferric reducing/antioxidant power (FRAP), and total phenolic contents, respectively.

## 2. Materials and methods

### 2.1. Chemicals

Tryptic soy agar (TSA), tryptic soy broth (TSB), dimethyl sulfoxide (DMSO), butylated hydroxytoluene (BHT), HCl, carbonate (Na_2_CO_3_), and sodium acetate (C_2_H_3_NaO_2_) were obtained from Merck (Darmstadt, Germany). Gallic acid, catechin, 2,4,6-Tris (2-pyridyl)-s-triazine (TPTZ), Trolox, FeCl_3_.6H_2_O, tris (hydroxymethyl) aminomethane were supplied from Sigma-Aldrich (Steinheim, Germany). Folin-Ciocalteu reagent, 2,2-diphenyl-1-picrylhydrazyl (DPPH) Fluka (St. Louis, MO, USA). Ofloxacin (OFX), netilmicin (NET30), and sulbactam (SCF) were obtained from Oxoid (Basingstoke, UK). All chemicals and solvents used were of analytical grade.

### 2.2. Extraction of CEO

Chrysopogon zizanioides (L.) Roberty (Syn. Vetiveria zizanioides (L.) Nash) was supplied from the field of İl-Ca Herbal Products Research-Development Production Company located in Giresun/Turkey in the Black Sea Region (40.979955, 38.783850) in September 2018. Dr. Mustafa KARAKÖSE identified and authenticated the plant (vetiver) material. The voucher specimen has been deposited (catalog number KATO: 16800) at the herbarium of the Faculty of Forestry Karadeniz Technical University (KATO), Trabzon/Turkey. Three-years old vetiver roots were washed many times and dried in the shade. A total of 150 g of the ground roots were subjected to hydrodistillation for 24 h in Clevenger apparatus (Thermal Laboratory Equipment, City?, Turkey). The extracted oil was dried with anhydrous sodium sulfate, filtrated, and then dissolved in 10% dimethyl sulfoxide (DMSO) to a final concentration of 1000 μg/mL and stored at +4 °C for further use. The yield of oil extraction was defined as mg/100 g dry vetiver root. The yield was determined as 0.88% (v/w).

### 2.3. Gas chromatography-mass spectrometry analyses of CEO

Analyses were performed using with Shimadzu GCMS-QP2010 system (Shimadzu Corporation, Kyoto, Japan). Analytical separations were performed using the Restek Rxi-5ms (Restek Corporation, Bellefonte, PA, USA), 5% diphenyl 95% dimethyl polysiloxane (30.0 m, 0.25 mm ID, 0.25 μm df) column and Supelco 50/30 um SPME fiber assembly divinylbenzene/carboxen/polydimethylsiloxane, Stableflex 24 Ga, manual holder, 3 pk (Supelco, Bellefonte, PA, USA). The oven temperature was set 40 °C, injection temperature was 250 °C and the final temperature was set to 240 °C to 40 °C. Ion source temperature was 210 °C and the interface temperature was 250 °C. The sampling time was 1 min. GC program time was 45 min. The pressure was set at 79.7 kPa. The ionization mode was at 70 eV. Scan mass range 40–400 amu. 1.44 mL/min helium gas was used as carrier gas and the split ratio was set at 1:25. The components were determined by comparison of their relative retention times and mass spectra with those of standards available on Wiley and NIST mass spectral libraries.

### 2.4. Antimicrobial activity of CEO

CEO was tested against Acinetobacter baumannii (ATCCBAA747), Klebsiella pneumoniae (ATCC13883), Citrobacter freundii (ATCC43864), Enterobacter aerogenes (ATCC3048), Pseudomonas aeruginosa (ATCC9027), Proteus mirabilis (ATCC43071), Bacillus megaterium (DSM32), Staphylococcus aureus (ATCC25923), Bacillus cereus (ATCC10876), Methicillin-resistant Staphylococcus aureus (MRSA) ATCC 67101, Bacillus subtilis (ATCC6633), Staphylococcus epidermidis (ATCC12228), Candida albicans (ATCC10231). The microorganisms were supplied from the Culture Collection of Erzurum Technical University (Erzurum, Turkey). The antimicrobial activity of the CEO was tested by the disc diffusion method and minimal inhibitory concentrations (MIC) were determined according to the microdilution method described before [9]. CEO was diluted with DMSO (10%) ranged between 31.5 and 700 μg/μL. For disc diffusion method, ofloxacin (OFX) (10 μg/disc), netilmicin (NET30) (30 μg/disc), sulbactam (SCF) (30 kg/disc) were used as positive references. For minimal inhibitory concentration determinations, maxipime (Bristol-Myers Squibb) in concentration between 500 and 7.81 μg/μL was used as a positive reference. A total of 10 μL of DMSO (10%) was a negative reference. Each experiment was performed three times.

### 2.5. Determination of total phenolic contents by Folin-Ciocalteu method

Total phenolic contents of CEO were determined by Folin-Ciocalteu reagent with some modifications using gallic acid and catechin as standard according to Slinkard and Singleton method [11]. The CEO concentration of 2.6 μg/mL was studied as turbidity occurred at high concentrations. Sample solution and standards (gallic acid and catechin) (50 μL) were diluted with distilled water (2.5 mL). A total of 250 μL Folin-Ciocalteu reagent (0.2 N) was mixed with the samples to be tested and then vortexed. After 3 min, 750 μL of Na_2_CO_3_ (7.5%) was added to the above solutions and vortexed again. After incubation at room temperature for 2 h, the sample absorbance was read at 765 nm. Each sample and standard concentration were run in 3 parallel. In addition, sample and reagent blank were studied for each concentration of each sample and standard. Total phenolic contents were expressed as μg gallic acid or catechin equivalent per mL sample [11,12].

### 2.6. Ferric reducing/antioxidant power (FRAP) method

The method used in this study is based on the measurement of the absorbance given by the later developed TPTZ-Fe (II) complex [13,14]. The activity of the CEO was determined by the calibration graph obtained using Trolox in the range of 31.25–1000 μM and the micromolar TEAC (Trolox equivalent antioxidant capacity) was determined. The essential oil was studied by diluting 1:24. In the method, a 50 μL sample was mixed with 1.5 mL of FRAP reagent (containing acetate buffer, TPTZ, and FeCl_3_.6H_2_0 solutions) and the absorbance was measured at 595 nm after 20 min incubation at room temperature. The pure water was considered as blank. CEO used in the FRAP method was analyzed from the stock by diluting 1:24 (41.67 μg/mL).

### 2.7. DPPH• radical scavenging activity

Radical scavenging activity was tested using the commonly used DPPH• radical agent [15]. A total of 750 μL of the samples were vortexed with 100 μM methanolic DPPH• solution in an equal volume (750 μL) and incubated at room temperature for 50 min. At the end of the period, the absorbance of the samples was read at 517 nm, where DPPH• gave maximum absorbance. All the experiments were performed thrice. The concentrations corresponding to the absorbance found were plotted and the IC_50_ values were calculated in μg/mL. Low IC_50_ values indicate higher radical cleaning potential. CEO was diluted with 1:24 dilution.

## 3. Results and discussion

### 3.1. GC-MS analyses

Qualitative and quantitative results obtained from GC-MS analysis are given in Table 1. As a result of the analysis, a total of thirty components were identified. The obtained major components were isobutyl-angelate (11.15%), α-muurolene (10.56%), α-cedrene (8.42%), α-patchoulene (8.10%), ethylene brassylate (7.49%), 2-methyl-undecanal (5.90%), α-bulnesene (5.58%), spathulenol (5.11%), α-gurjunene (3.51%), and methyl-palmitate (3.36%).

The essential oil of Chrysopogon zizanioides (L.) Roberty roots have aromatic characteristics due to its content [16,17]. This plant has been cultivated all over the world. As known, the chemical compositions of the essential oils can vary depending on the geographical region and climate characteristics of the region. Therefore, it was decided to determine the chemical composition of essential oil obtained from the roots of Chrysopogon zizanioides (L.) Roberty grown in Giresun. According to the literature, CEOs contain vetiselineol, α-vetivone, β-vetispirene, and khusimol as major compounds, which are characteristics of vetiver [3,18]. However, in this study, none of the major components of CEOs were determined. It may be due to the differences in reference compounds registered in the GC-MS library used. Some compounds determined in this study were α-muurolene (10.56%), spathulenol (5.11%), α-copaene (2.93%), γ-cadinene (1.76%), 2,4-dimethyl-acetophenone (1.08%) which comply with the previous reports [19–23]. However, these compounds were found to be higher in this study compared to the other studies in the literature (Table 2). The amount of α-bulsene, α-amorphene, α-curcumene and aromadendrene were found to be less in this study compared to the other studies in the literature [19–24] (Table 2). In addition, α-gurjunene and β-patchoulene were found to be in approximate ratios compared to those found in the literature [19,20,25]. Differently, isobutyl-angelate, α-cedrene, α-patchoulene, ethylene brassylate, 2-methyl-undecanal, methyl-palmitate, palmitic acid, β-cedrene, tetrahydro furfuryl propionate, etc. found in the essential oil of vetiver root used in this study were not reported in any vetiver essential oil. It was concluded there were significant differences in terms of the secondary metabolites produced by the plant grown in different regions. Due to its different active compounds, it was clear that the biological activities of the CEO (antimicrobial and antioxidant activities) would exhibit differences [17].

### 3.2. Antimicrobial activity

The results of antimicrobial activity were given in Table 3. MIC values of CEO were 31.25 μg/disc for C. albicans, 62.5 μg/disc for A. baumannii, E. aerogenes, S. aureus, B. cereus, B. subtilis, 125 μg/disc for K. pneumonia, C. freundii, P. aeruginosa, P. mirabilis, B. megaterium, MRSA, S. epidermis, respectively. As seen in Table 3, the CEO showed great antimicrobial activity against all of the tested clinical pathogens without any significant difference in susceptibility between gram-positive and gram-negative.

Several types of research have reported essential oil of vetivers grown in different geographical regions to have antibacterial activity against bacterial strains including Staphylococcus aureus, Streptococcus pyogenes, Escherichia coli, Corynebacterium ovis, Mycobacterium smegmatis, Acinetobacter baumannii, Aeromonas veronii, Klebsiella pneumoniae, P. aeruginosa, Salmonella enterica, Serratia marcescens, Enterococcus faecalis, Enterebacter cloacae and Proteus vulgaris [3,26–28]. In addition, there have been many studies that indicated the antifungal activity of CEO against Alternaria alternate, Fusarium oxysporium, Rhizoctonia bataticola, Sclerotium rolfsii, Aspergillus niger, Candida albicans, and Cryptococcus neoformens [3,25,26,29]. The results of this study are in compliance with the previous reports. Consequently, the significant antimicrobial activity of the CEO and the differences in the level of antimicrobial activities between this study and previous studies could be attributed to the contents of CEOs. According to the results of this study, it was observed that CEO showed inhibition effect more than antibiotics or almost as much as antibiotics against tested microorganisms at high concentrations of CEO. Antibiotics are the most commonly used medicines [9]. Besides their common utilization, these compounds are also unconsciously used according to the official records [9]. However, antibiotics have serious side effects on the ecosystem and all the organisms in an ecosystem. The increasing antibiotic resistance threaten human health as common infections have become much more difficult to treat [30]. Therefore, there is a significant need for scientific innovation for new antimicrobials without any side effects. According to the results of this study, the CEO has a great potential to use as an antimicrobial agent instead of antibiotics.

### 3.3. Antioxidant activity

Antioxidants from natural sources are molecules, which act as free radical scavengers. They have a preventive effect oxidative reaction that leads to various diseases. In this study, three different antioxidant activity assays based on different reaction mechanisms were used to detect the potent antioxidant activity of CEO; DPPH, FRAP, and total phenolic contents. According to the DPPH results, CEO showed strong free radical scavenging activity compared to the butylated hydroxytoluene (BHT) standard antioxidant (IC_50_ 7.124 ± 0.076 μg/mL) (Table 4). However, it was about 1.92 times less radical cleaning activity than the Trolox standard. Also, the methods of FRAP and total phenolic content determination of CEO were applied for the first time. FRAP value of the sample was found to be 356.44 ± 2.34 μM TEAC. Thus, antioxidants found in CEO could inhibit electron transfer by forming a complex with iron +3 ions. The total phenolic content of the essential oil was found to be 13.70 ± 2.10 μg/mL GAE and 24.67 ± 3.79 μg/mL CE. Recently, total phenolic content of vetiver root has been reported but not essential oil obtained from vetiver roots [27,31]. The determination of antioxidant activity with FRAP is almost very limited [32]. Hence, in this study, the CEO was investigated using in vitro assays including FRAP and total phenolic content. According to the literature, the high antioxidant activity of CEOs might be related to their major constituents and natural active compounds of CEO. Therefore, there has been an increasing demand for CEOs to be used in the medicine and food industry [3,5].

## 4. Conclusion

In this study, the chemical composition and bioactivities of the essential oil obtained from the roots of Chrysopogon zizanioides (L.) Roberty cultivated in Giresun, Turkey were determined. According to the data from World Health Organization, nearly 6 million people die due to lack of access to existing antibiotics annually. Chrysopogon zizanioides (L.) Roberty can be cultivated all over the world and its essential oil can be used as alternative antimicrobial agents. The antioxidant results of the CEO indicate that the essential oil has better antioxidant activity than BHT. Besides, CEO is a good candidate to use as an antimicrobial. Thus, CEO can be used both as an antimicrobial and antioxidant.

## Figures and Tables

**Table 1:** The composition of essential oil of Chrysopogon zizanioides roots.

Compound name	Area (%)	RI
Capronaldehyde	1.31	747
Phenylacetaldehyde	0.82	766
Octa-2(E),4(E)-dienal	1.16	860
2,4-dimethyl-acetophenone	1.08	897
α-Curcumene	1.75	1353
Cyclosativene	2.27	1374
α-Cedrene	8.42	1390
α-Copaene	2.93	1399
Elemene δ->	1.62	1420
β-Cedrene	1.91	1422
α-Gurjunene	3.51	1434
Cadina-1(6),4-diene	1.59	1442
α-Bulnesene	5.58	1457
α-Patchoulene	8.10	1462
α-Muurolene	10.56	1490
α-Amorphene	1.88	1496
Spathulenol	5.11	1501
γ-Cadinene	1.76	1505
β-Patchoulene	2.73	1532
Jasmone	0.74	1548
Tetrahydrofurfuryl propionate	1.46	1607
Aromadendrene	0.85	1613
Ethylene brassylate	7.49	1644
Anethofuran	0.82	1680
Cadina-1(6),4-diene	0.51	1697
methyl-Myristate	1.40	1715
2-methyl-Undecanal	5.90	1911
methyl-Palmitate	3.36	1924
Palmitic acid	2.21	1950
isobutyl-Angelate	11.15	2313

* Retention indices (RIs) relative to n-alkanes (C_7_-C_30_) on the same capillary column.

**Table 2:** Comparation of GC-MS analyses of different studies.

Compounds	Present study	[18]	[19]	[20]	[21]	[22]	[23]
Capronaldehyde	1.31	-	-	-	-	-	-
Phenylacetaldehyde	0.82	-	-	-	-	-	-
Octa-2(e),4(e)-dienal	1.16	-	-	-	-	-	-
2,4-dimethyl-acetophenone	1.08	-	Trace levels	-	-	-	-
α-curcumene	1.75	-	0.2–4.3	11.92	0.6	0.11–2.72	2.44
Cyclosativene	2.27	-	-	-	-	-	-
α-cedrene	8.42	-	-	-	-	-	-
α-copaene	2.93	-	-	0.12–1.04	-	-	-
Elemene δ->	1.62	-	7.2–12.7	-	-	-	-
β-cedrene	1.91	-	-	-	-	-	-
α-gurjunene	3.51	-	4.2–9.8	1.02–1.38	-	-	5.91
Cadina-1(6),4-diene	1.59	-	-	-	-	-	-
α-bulnesene	5.58	-	7.1	-	-	-	-
α-patchoulene	8.10	-	-	-	-	-	-
α-muurolene	10.56	-	-	2.46–3.4	-	-	-
α-amorphene	1.88	-	7.8–8	3.54	0.26	-	7.80
Spathulenol	5.11	-	0.3–7.3	-	0.2	-	2.47
γ-cadinene	1.76	-	Rich cadiene types	0.12–18	0.26	0.49–0.53	-
β-patchoulene	2.73	-	-	0.92–3.15	-	-	-
Jasmone	0.74	-	-	-	-	-	-
Tetrahydrofurfuryl propionate	1.46	-	-	-	-	-	-
Aromadendrene	0.85	-	0.3–6.9	-	7.34–9.66	7.34–9.66	5.45
Ethylene brassylate	7.49	-	-	-	-	-	-
Anethofuran	0.82	-	-	-	-	-	-
Cadina-1(6),4-diene	0.51	-	-	-	-	-	-
Methyl-myristate	1.40	-	-	-	-	-	-
2-methyl-undecanal	5.90	-	-	-	-	-	-
Methyl-Palmitate	3.36	-	-	-	-	-	-
Palmitic acid	2.21	-	-	-	-	-	-
Isobutyl-Angelate	11.15	-	-	-	-	-	-

**Table 3:** Antimicrobial activity of the essential oil of Chrysopogon zizanioides roots.

Bacteria	Concentrations (μg/μL)							MIC***	Negative control**	Standard antibiotic discs		
	700	600	500	250	125	62.5	31.25			OFX*	NET30	SCF
A. baumannii	17	15	14	10	8	7	-	62.5	-	21	16	16
K. pneumoniae	14	13	12	9	8	-	-	125	-	18	14	15
C. freundii	20	18	14	10	7	-	-	125	-	25	24	21
E. aeuriginosa	20	18	14	10	9	7	-	62.5	-	20	21	19
P. aeruginosa	16	15	15	10	7	-	-	125	-	19	20	18
P. mirabilis	19	16	14	10	7	-	-	125	-	21	20	17
B. megaterium	22	19	17	12	8	-	-	125	-	20	19	16
S. aureus	22	20	19	15	10	8	-	62.5	-	20	20	17
B. cereus	23	23	20	15	9	7	-	62.5	-	20	20	19
MRSA ATCC 67101	20	17	13	8	8	-	-	125	-	20	20	14
B. subtilis	22	20	18	15	11	7	-	62.5	-	20	20	21
S. epidermis	37	25	20	14		-	-	125	-	22	21	17
C. albicans	22	20	18	14	8	-	-	31.25	-	20	19	18

The positive controls were ofloxacin (OFX) 10 μg/disc, netilmicin (NET) (30 μg/disc), and sulbactam (SCF) (30 μg/disc). The negative control was dimethyl sulfoxide (DMSO) (10%). Zones of growth inhibition were measured in milimeter (mm). MIC (minimal inhibition concentration) was calculated as μg/mL.

**Table 4:** Total phenolic contents and antioxidant activity of Chrysopogon zizanioides roots essential oil and standards.

Sample and standards	*Total phenolic content (GAE and CE, μg/mL)	*DPPH• scavenging (IC_50_, μg/mL)	*FRAP (TEAC, μM)
CEO-GAE CEO-CE	13.70 ± 2.10 24.67 ± 3.79	7.124 ± 0.076	356.44 ± 2.34
BHT	NT	8.408 ± 0.186	NT
Trolox	NT	3.704 ± 0.005	NT

* All test results were presented as mean ± standard error (SD) in triplicate.

Chrysopogon zizanioides essential oil (CEO); gallic acid equivalent (GAE); catechin equivalent (CE); butylated hydroxytoluene (BHT); 2,2-diphenyl-1-picrylhydrazyl (DPPH•); ferric reducing power (FRAP); trolox equivalent antioxidant capacity (TEAC); not tested (NT).
